# Color Matching Ability of a Single‐Shade or Multi‐Shade Composite Used for Lithium Disilicate Restoration Repair: Influence of Background and Shade

**DOI:** 10.1155/ijod/2269070

**Published:** 2026-07-27

**Authors:** Fatemeh Pournaghi Azar, Haleh Valizadeh-Haghi, Safa Valizadeh-Haghi

**Affiliations:** ^1^ Department of Restorative Dentistry, Tabriz University of Medical Sciences, Tabriz, Iran, tbzmed.ac.ir; ^2^ Department of Restorative Dentistry, Ardabil University of Medical Sciences, Ardabil, Iran, arums.ac.ir; ^3^ Social Determinants of Health Research Center, Ardabil University of Medical Sciences, Ardabil, Iran, arums.ac.ir

**Keywords:** color matching, lithium disilicate ceramic, single-shade composite

## Abstract

**Introduction:**

In recent years, single‐shade composites have attracted considerable attention due to simplification of the shade selection process. This study aimed to evaluate the color matching ability of a single‐shade composite with different shades of lithium disilicate ceramic.

**Materials and Methods:**

In this in vitro study, 66 specimens were evaluated in six groups. Lithium disilicate ceramic discs in three shades (A1, A2, and A3) were fabricated with a central cavity and, depending on the group, filled with either a single‐shade composite (Essentia, GC) or a multi‐shade composite (Estellite Sigma Quick). Three solid ceramic discs with identical dimensions were prepared for each shade and served as color reference specimens. Color measurements were performed at the center of the discs using a spectrophotometer over black and white backgrounds, and color differences (Δ*E*_00_) were calculated relative to the reference specimens. Data were analyzed using three‐way ANOVA. Subgroup comparisons were performed using one‐way ANOVA followed by Tukey’s HSD post hoc test. The level of significance was set at 5%.

**Results:**

Three‐way ANOVA demonstrated significant effects of ceramic shade and background color on color‐matching ability (*p*  < 0.001). Significant interactions were observed among composite type, ceramic shade, and background condition (*p*  < 0.001). The single‐shade composite demonstrated superior color matching with A3 ceramics under both background conditions, whereas the multi‐shade composite showed better performance with A1 ceramics.

**Conclusion:**

The color‐matching ability of repair composites was significantly influenced by the interaction among composite type, ceramic shade, and background color.

## 1. Introduction

Single‐shade composites were introduced to dentistry with the claim of having the ability to adapt to and reflect the color of the surrounding structure [1]. Among the advantages of these materials are the elimination of the need for shade selection during restorative procedures, which may reduce chair time, eliminate the need for advanced shade‐matching skills, and obviate the requirement to store multiple composite shades in dental offices [2]. According to manufacturers’ recommendations, the indications for these composites include anterior and posterior direct restorations, direct composite veneers, diastema closure, and the repair of composite and ceramic restorations [3, 4].

In recent years, numerous studies have investigated the color‐matching ability of single‐shade composites, most of which have been conducted on natural teeth, acrylic teeth, or composite substrates. Conflicting results have been reported across studies; some studies demonstrated superior color matching of single‐shade composites compared with conventional multi‐shade composites, while others reported comparable performance or even clinically unacceptable color matching for single‐shade composites [5–11].

However, considering that one of the applications of these composites is as repair materials for ceramic restorations, only limited studies have investigated the color‐matching ability of single‐shade composites with dental ceramics, particularly the widespread lithium disilicate ceramics [11–14]. On the other hand, there is a growing trend toward direct intraoral repair of ceramic restorations rather than complete removal and replacement [15]. Factors such as inadequate thickness, fatigue loading, inherent porosities within the ceramic structure, and mismatched coefficients of thermal expansion may contribute to ceramic restoration fractures [16]. Direct intraoral repair preserves both tooth and restoration structure, prevents unnecessary removal of sound tissues, and reduces additional trauma [15].

In addition to bonding effectiveness, which has been extensively studied, the optical properties of the repair composite are critical to the esthetic outcome of repaired ceramic restorations [15, 17, 18]. Successful optical integration depends on multiple factors, including shade compatibility between the repair composite and ceramic substrate, translucency of both materials, refractive index compatibility, material thickness, substrate reflectance, and background color [12]. In clinical situations, restorations placed at the incisal edge or over discolored tooth structures may therefore exhibit different optical behavior compared with restorations placed over normal dentin structures [19]. Because single‐shade composites rely more heavily on structural color and light transmission mechanisms than conventional multi‐shade composites, their final perceived color may be strongly influenced by the optical behavior of the surrounding structure and underlying background [20]. Additionally, the interactions with the underlaying background may become particularly important in lithium disilicate ceramics due to their relatively high translucency and light‐transmitting properties [21, 22].

Given the inconsistent findings reported in previous studies and the limited evidence regarding the optical behavior of single‐shade composites in ceramic repair situations, the aim of the present study was to assess the color‐matching ability of a single‐shade composite used for simulated intraoral repair of lithium disilicate ceramics with different shades under different background conditions and to compare its performance with that of a multi‐shade nanohybrid composite. The null hypothesis was that the composite type, ceramic shade, and background color would not significantly affect the color‐matching ability of the tested materials.

## 2. Materials and Methods

This in vitro study was performed on lithium disilicate ceramic discs, and two composite materials were used to simulate the direct intraoral repair of ceramic restorations. IPS e.max Press lithium disilicate ceramic (Ivoclar Vivadent, Liechtenstein) in A1, A2, and A3 shades with medium translucency was selected as the substrate material. Simulated ceramic repair was carried out under laboratory conditions using either a single‐shade composite (Essentia, GC, Japan) or a multi‐shade nanohybrid composite (Estelite Sigma Quick, Tokuyama, Japan) in the corresponding shades.

The sample size calculation was based on a similar study [9]. Considering the reported mean ± standard deviation of color changes in that study (4.52 ± 0.32 and 10.3 ± 0.88), with a statistical power of 0.90 and a type I error of 0.05, the sample size was calculated using 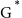
Power software. A minimum of 11 specimens per group was determined.

**Figure 1:**
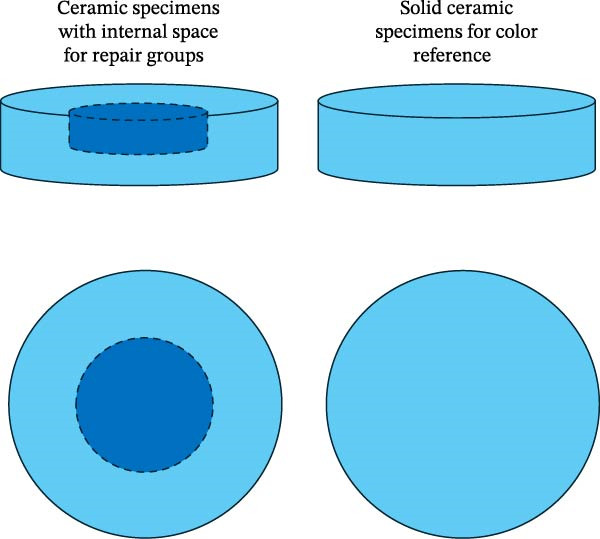
Schematic view of the ceramic specimens.

For each ceramic shade, three specimens were prepared as color reference samples. These reference specimens consisted of discs with a diameter of 8 mm and a thickness of 2 mm in shades A1, A2, and A3 with medium translucency. For the repair specimens, ceramic discs with identical dimensions (8 mm diameter and 2 mm thickness) were fabricated in the same three shades, with a centrally located cylindrical cavity measuring 4 mm in diameter and 1 mm in depth. The cavity dimensions were selected according to the diameter of the spectrophotometer tip to allow a standardized and reliable color measurement from the central repair area while preserving the surrounding ceramic structure. This cavity was later filled with the repair composite material. Fabrication of the ceramic discs was performed by a prosthetic technician according to the manufacturer’s instructions using the lost‐wax technique. To minimize variability, the molds were designed and fabricated using CAD/CAM technology. All ceramic discs were glazed except for the central cavity area, which simulated the fractured region. A schematic illustration of the reference and repair specimens is shown in Figure 1.

For the repair specimens, ceramic surfaces were conditioned prior to composite application to simulate clinical conditions. The internal surface of the cylindrical cavity in each ceramic disc was etched with 9% hydrofluoric acid (Cera‐Etch, Morvabon, Iran) for 20 s, followed by rinsing and drying. To minimize the influence of an additional optical variable, no bonding agent was applied. Instead, a thin glycerin layer was used to partially simulate the optical effect of the adhesive interface, as described in a previous study [11].

The cavity was then filled with the assigned composite material in a single increment using a spatula. To obtain a flat surface, a celluloid strip was placed over the composite‐filled cavity, and the assembly was manually pressed between two glass slides. Light curing was performed for 40 s using a single‐point irradiation technique, with the light‐curing tip positioned perpendicular and as close as possible to the specimen surface. A light‐curing unit (Radii Plus, SDI, Australia) with a manufacturer‐reported irradiance of 1500 mW/cm^2^ was used. Before specimen preparation, the device output was checked using the built‐in light intensity indicator provided by the manufacturer to ensure an adequate irradiance output.

After polymerization, the specimen thickness was verified using a digital caliper. Specimens deviating more than 0.05 mm from the intended dimensions were discarded and remade. No additional finishing or polishing procedures were performed as the celluloid strip provided a standardized smooth surface for all specimens. Specimens were stored in distilled water at 37°C for 24 h to allow completion of early postpolymerization reactions prior to color measurement [15].

In the single‐shade composite group, Essentia (GC, Japan) was used for all ceramic discs. In the multi‐shade composite group, given that the ceramic discs had medium translucency, Estelite Sigma Quick composites with medium opacity and the corresponding shades were selected. Accordingly, OA1, OA2, and OA3 composites were used for A1‐, A2‐, and A3‐shaded ceramics, respectively. Initial pilot evaluation using A1, A2, and A3 shades of Estelite Sigma Quick composites revealed visually noticeable color mismatches; therefore, the corresponding medium‐opacity (OA) shades were selected to achieve better color integration with the ceramic substrate.

Color measurements were performed using a YS3020 diffuse/8° spectrophotometer (3nh, China). Measurements were taken precisely at the center of each disc—corresponding to the ceramic area in reference specimens and the composite area in repair specimens—over white (L^∗^ = 92.62, a^∗^ = −0.91, b^∗^ = 5.04) and black (L^∗^ = 25.68, a^∗^ = −0.33, b^∗^ = −0.21) backgrounds. Color coordinates were recorded according to the CIE *Lab* ^∗^ color system. Each specimen was measured three times, and the mean of the recorded values was used for statistical analysis.

To assess color‐matching ability, the color difference between repaired specimens and the mean color coordinates of the corresponding reference groups was calculated using the CIEDE2000 color‐difference formula (Δ*E*_00_) [9].

ΔE00=ΔL′/K_LS_L2+ΔC′/K_CS_C2+ΔH′/K_HS_H2+ R_TΔC′/K_CS_CΔH′/K_HS_H12/.

According to Paravina et al. [23], Δ*E*_00_ values below 0.8 were considered clinically imperceptible, whereas values above 1.8 were considered clinically unacceptable.

Data distribution was assessed using the Shapiro–Wilk test, and the homogeneity of variances was evaluated using Levene’s test. Although some deviation from normality was observed, parametric analysis was considered appropriate due to the balanced design and equal sample sizes across groups. Three‐way ANOVA was used to evaluate the effects of composite type, ceramic shade, and background color, as well as their interactions, on color‐matching ability. Effect sizes were calculated using partial eta squared (ηp^2^). Because a significant three‐way interaction was observed, one‐way ANOVA followed by Tukey’s HSD post hoc test was performed for subgroup comparisons among the tested groups. The significance level was set at *p*  < 0.05 for all analyses.

## 3. Results

**Figure 2:**
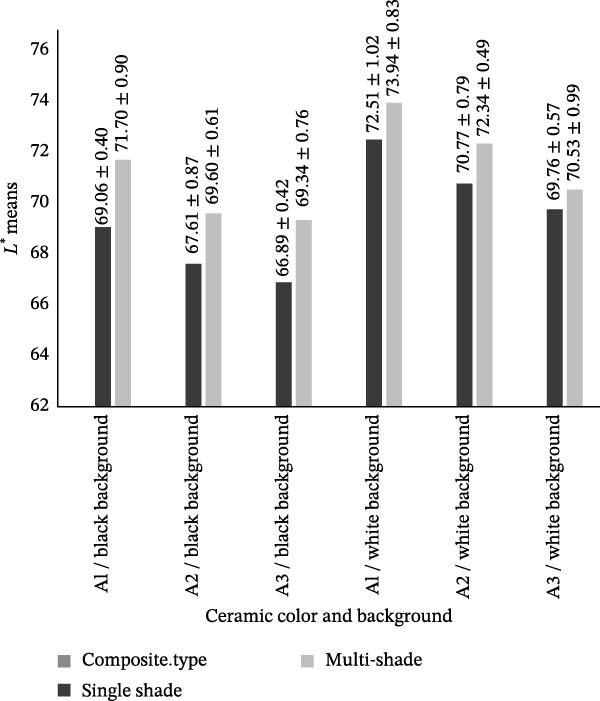
*L* ^∗^ values of different groups.

**Figure 3:**
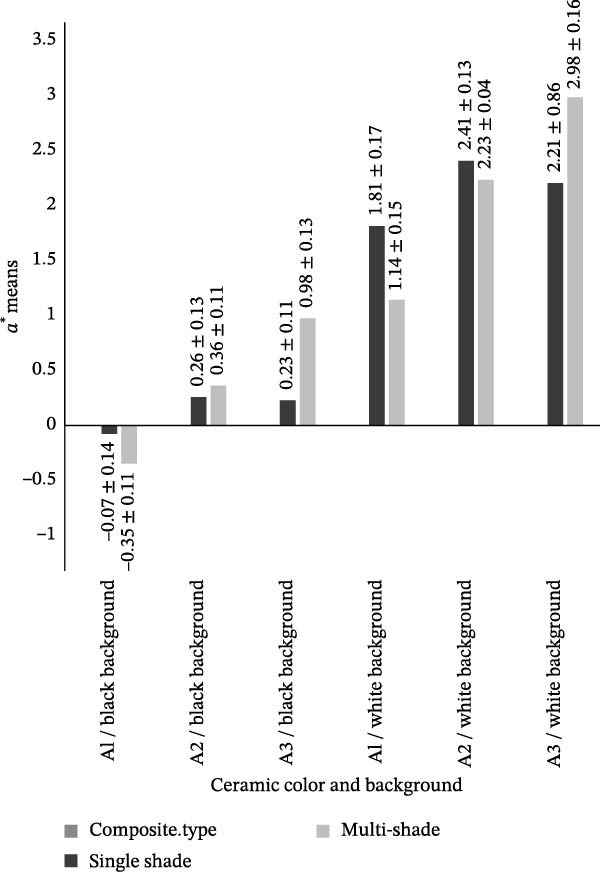
*a* ^∗^ values of different groups.

**Figure 4:**
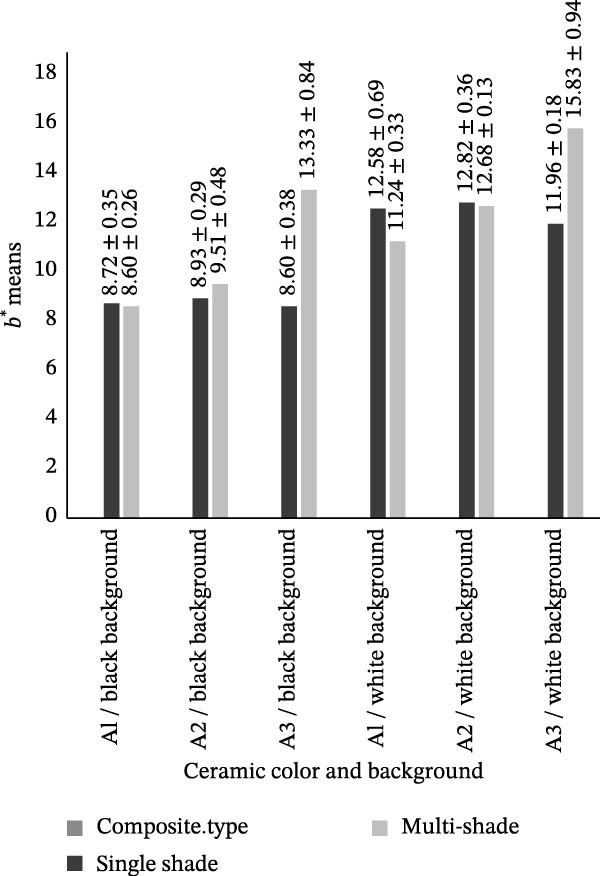
*b* ^∗^ values of different groups.

**Figure 5:**
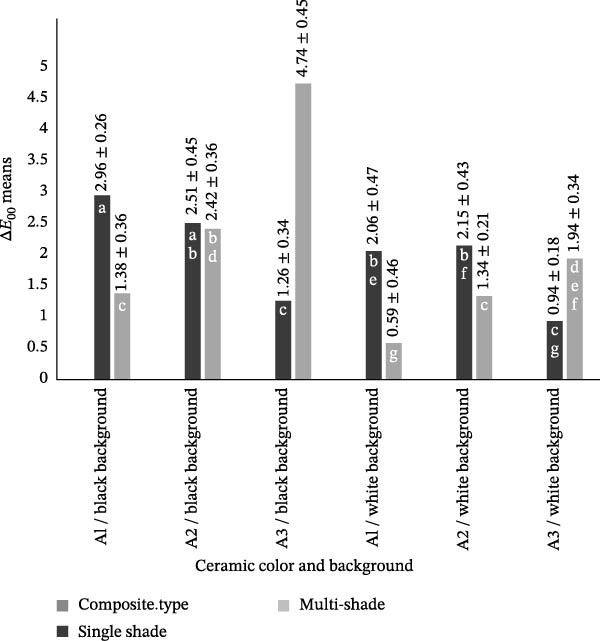
The mean values of ∆*E*_00_ in different groups. Groups indicated by the same letters were not significantly different statistically.

The mean values of the color coordinates measured for the reference specimens under different background conditions are presented in Table 1. Descriptive values of the color coordinates (L^∗^, a^∗^, and b^∗^) of the tested groups, as well as the mean Δ*E*_00_ values, are presented in Figures 2–5.

**Table 1:** Color coordinates of the reference ceramic discs.

Background	Ceramic shade	Mean (SD) of L^∗^	Mean (SD) of a^∗^	Mean (SD) of b^∗^
Black	A1	72.36 (0.06)	−0.76 (0.06)	7.20 (0.03)
	A2	69.50 (0.02)	−0.39 (0.02)	6.60 (0.04)
	A3	65.42 (0.01)	0.02 (0.01)	8.41 (0.02)
White	A1	74.26 (0.36)	0.99 (0.04)	11.00 (0.03)
	A2	72.86 (0.10)	1.95 (0.03)	10.80 (0.06)
	A3	72.69 (0.35)	2.59 (0.15)	13.16 (0.14)

The Shapiro–Wilk test indicated some deviation from normality in the Δ*E*_00_ values, whereas Levene’s test confirmed homogeneity of variances among the groups (*p* = 0.729). Three‐way ANOVA demonstrated that ceramic shade and background color significantly affected color‐matching ability (*p*  < 0.001). Significant two‐way interactions were also observed between composite type and ceramic shade (*p*  < 0.001), composite type and background color (*p*  < 0.001), and ceramic shade and background color (*p*  < 0.001). Furthermore, a significant three‐way interaction was observed among composite type, ceramic shade, and background color (*p*  < 0.001). Detailed results of the three‐way ANOVA and effect size analyses are presented in Table 2.

**Table 2:** The results of three‐way ANOVA test.

Source	Type III sum of squares	*df*	Mean square	*F*	Sig.	Partial eta squared
Corrected model	147.262^a^	11	13.387	96.678	0.000	0.899
Intercept	541.338	1	541.338	3909.260	0.000	0.970
Composite.type	0.258	1	0.258	1.860	0.175	0.015
Ceramic.color	5.384	2	2.692	19.439	0.000	0.245
Background	35.953	1	35.953	259.631	0.000	0.684
Composite.type ^∗^ ceramic.color	82.710	2	41.355	298.644	0.000	0.833
Composite.type ^∗^ background	8.843	1	8.843	63.859	0.000	0.347
Ceramic.color ^∗^ background	4.584	2	2.292	16.552	0.000	0.216
Composite.type ^∗^ ceramic.color ^∗^ background	9.532	2	4.766	34.416	0.000	0.365
Error	16.617	120	0.138			
Total	705.217	132				
Corrected total	163.880	131				

^a^R Squared = 0.899 (Adjusted R Squared = 0.889).

Because significant interaction effects were identified, subgroup comparisons were performed using one‐way ANOVA followed by Tukey’s HSD post hoc test (*p*  < 0.001). The results of these comparisons are summarized in Figure 5.

## 4. Discussion

In the present study, the color‐matching ability of a single‐shade composite used for simulated direct repair of lithium disilicate ceramics with different shades was evaluated and compared with that of a multi‐shade composite. The findings demonstrated a significant interaction among composite type, ceramic shade, and background color on color‐matching ability; therefore, the null hypothesis was rejected.

Previous studies investigating the color‐matching ability of single‐shade composites have reported conflicting results. Several investigations demonstrated inferior color‐matching outcomes for single‐shade composites compared with multi‐shade composites [24–30], whereas Kobayashi et al. [10] in 2021, Pereira et al. [31] in 2019, Saegusa et al. [32] in 2019, and Chen et al. [33] in 2021 reported superior performance for single‐shade composites. These inconsistencies may be attributed to methodological differences and variations in the materials used among studies. The findings of the present study support this assumption as a significant interaction was observed among composite type, ceramic shade, and background, indicating that the color‐matching behavior of single‐shade composites cannot be explained by a single factor alone. Therefore, not only the restorative material itself but also the optical characteristics of the substrate and surrounding background appear to influence the final color outcome. Different studies have used various substrates, including natural teeth, acrylic teeth, composite specimens, and ceramic materials, all of which may influence the reported results.

Additionally, it is noteworthy that in most previous studies evaluating the color‐matching ability of single‐shade composites, background conditions were either not specified or not considered an experimental variable [10, 24–31]. This may be related to methodological differences and the use of bulky specimens such as extracted teeth, acrylic teeth, or composite artificial teeth, in which the influence of the underlying background is reduced. Caliskan et al. [11], who evaluated disc‐shaped specimens, placed a 4‐mm‐thick composite block beneath the samples to minimize background influence and reported clinically unacceptable color matching for single‐shade composites used as repair materials. Similarly, Genger et al. [13], in their comparison of two single‐shade composites for ceramic repair, used a neutral gray background to reduce background effects and observed color differences exceeding the clinically acceptable threshold. In contrast, Barros et al., who used a white background in their study, recommended that future investigations incorporate darker backgrounds for a more comprehensive optical evaluation [7].

Studies have demonstrated that background color significantly influences the final appearance of restorative materials, particularly in highly translucent materials [34]. Furthermore, in clinical situations, ceramic restorations are often relatively thin; therefore, the optical properties of the underlying structure can considerably influence the optical outcome of the repaired restorations. Factors such as metallic substructures, discolored tooth structure beneath the restoration, or the dark appearance of the oral cavity visible through thin incisal areas or through‐and‐through restorations may significantly affect the final perceived color [21, 22] and consequently the color‐matching performance of repair composites [19]. When specimens are placed on a white background, reflected light becomes more uniform, reducing the perception of certain visible‐spectrum reflections and thereby producing a more colorless appearance [32]. The findings of the present study support the importance of this factor as most tested groups demonstrated significantly better color matching on the white background compared with the black background. The exceptions were the single‐shade composite used with A2 and A3 ceramics, in which the background did not significantly affect color‐matching ability.

In the present study, under both black and white background conditions, the single‐shade composite demonstrated better color matching than the multi‐shade composite with the A3 ceramic, whereas the opposite relationship was observed with the A1 ceramic. These results contrast with the findings of Habib et al. [5], Barros et al. [7], Iyer et al. [8], and Islam et al. [9], in which single‐shade composites exhibited superior color matching with lighter shades. Iyer attributed this phenomenon to higher light transmission in lighter shades, which may enhance color blending [8]. Conversely, some studies reported no significant difference in the color‐matching ability of single‐shade composites across different shades. In these studies, the high translucency of the materials was cited as the reason for uniform color matching across shades [6, 24]. Ercin et al. evaluated the color matching of several single‐shade composites on acrylic teeth in shades A1, A2, and A3 over a black background and found that all composites exhibited the smallest color difference with A3 [35], which is consistent with the present findings. The observed discrepancies among studies may be attributed to the differences in methodology. Barros demonstrated that the blending effect of the single‐shade composite Unique APS Vittra was more pronounced with lighter shades; however, this study only used a white background, and darker backgrounds may yield different results. Additionally, sample thickness affected color matching, such that at 1 mm thickness, A3 demonstrated better blending than A1, whereas at 1.5 mm thickness, no difference was observed [7].

Furthermore, most previous studies evaluated Omnichroma as the single‐shade composite, which may behave differently from the Essentia composite used in the present study as Omnichroma relies on structural color technology and does not contain conventional dyes or pigments [33]. In contrast, Essentia combines high translucency and light transmission properties with the presence of pigments [24], which may explain the present findings as pigments can improve color matching with higher‐chroma ceramics. Paolone et al. also stated that the use of specific color pigments may enhance the color compatibility of resin composites [12]. Collectively, these findings indicate that the blending effect is a complex phenomenon influenced not only by the composite itself but also by factors such as cavity size, restoration thickness, and background color [35]. Therefore, the color adaptation behavior of single‐shade composites appears to be material‐ and condition‐dependent rather than universally predictable.

In the present study, 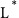 values were higher for both composites on the white background; however, the multi‐shade composite demonstrated higher 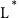 values under all tested conditions. The 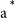 and 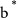 values were also generally higher on the white background for both composites, although the differences between the two composite types varied depending on the ceramic shade. In A3‐shaded ceramics, under both background conditions, the single‐shade composite exhibited lower 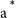 and 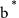 values than the multi‐shade composite, indicating lower redness and yellowness. In the study by Saegusa et al., which compared the effects of white and black backgrounds on the color characteristics of the single‐shade composite Omnichroma and conventional multi‐shade composites, it was concluded that the two types of composites exhibited different optical behaviors under different background conditions. 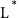 values were higher for all materials on the white background; however, the single‐shade composite demonstrated greater variation between backgrounds. In contrast, 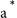 values were higher on the black background for multi‐shade composites, whereas the single‐shade composite showed higher 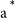 values on the white background. Furthermore, 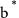 values were higher on the white background for all materials, although multi‐shade composites appeared to be more affected by background changes [32]. Taken together, these factors may influence the final color‐matching outcome. Furthermore, these findings highlight the importance of background conditions; however, their effects appear to be material‐dependent.

In the CIEDE2000 color‐difference system, the perceptibility threshold (PT) and acceptability threshold (AT) are clinically important parameters. According to previously proposed thresholds, Δ*E*_00_ values below 0.8 are considered imperceptible, whereas values above 1.8 are considered clinically unacceptable [36]. In the present study, Δ*E*_00_ values were below the PT only in the multi‐shade composite group with A1 ceramic over the white background, whereas all other groups exhibited perceptible color differences. Nevertheless, the single‐shade composite demonstrated clinically acceptable color differences with the A3 ceramic under both background conditions. In contrast, the multi‐shade composite showed acceptable color differences with the A1 ceramic on both backgrounds and with the A2 ceramic on the white background.

In theory, multi‐shade composites are expected to demonstrate superior color matching because shade selection can be performed according to the surrounding restoration. However, in the present study, the multi‐shade groups were standardized by using only medium‐opacity composites with shades corresponding to the ceramic substrate and applying them in a single layer. This methodology differs from clinical situations, in which clinicians may use a wider variety of composite systems, translucencies, and layering techniques to optimize color integration [37]. Therefore, the color discrepancies observed in some multi‐shade composite groups may partly be related to these methodological limitations, which could have influenced the final optical outcomes.

Other minor factors affecting the color matching of single‐shade composites have also been explored. Adhesive systems may influence the optical behavior of these materials because of their high translucency [33] and may therefore contribute to variations among studies. In the present study, adhesive systems were not applied to avoid introducing an additional optical variable. Instead, glycerin was used to simulate the optical properties of the adhesive layer and to eliminate air gaps between the composite and ceramic surfaces. Various materials, including optical gel, distilled water, oil, and glycerin, have been suggested for this purpose in dental research [11, 38]. Glycerin, with a refractive index similar to that of optical gel, has been used to enhance light transmission between materials [11, 36] and was therefore selected to simulate the bonding interface in the present study.

Finally, it should be noted that the present study was an in vitro investigation and could not completely reproduce clinical conditions. The study was performed on flat specimens, whereas natural teeth and restorations in the oral environment exhibit complex convex and concave surfaces that may influence the final color perception. In addition, due to resource limitations, it was not possible to evaluate different ceramic shades and translucencies, various cavity dimensions, different commercially available single‐shade composites, or long‐term thermomechanical aging protocols. Furthermore, glycerin could not fully reproduce the optical behavior of a polymerized adhesive layer, so the potential optical effect of the adhesive layer on the final color outcome was not investigated and may be considered in future studies.

## 5. Conclusion

Based on the results of this study and considering its limitations, the following conclusions can be drawn:

The color‐matching ability of repair composites was significantly influenced by the interaction among the composite type, ceramic shade, and background color.

The single‐shade composite demonstrated more favorable color matching with higher‐chroma ceramics, whereas the multi‐shade composite showed better performance with lighter ceramic shades.

## Funding

No funding was received for this manuscript.

## Conflicts of Interest

The authors declare no conflicts of interest.

## Data Availability

The data that support the findings of this study are available from the corresponding author upon reasonable request.
